# Impact of Point Prevalence Surveillance on Transmission of Candida auris Within an Acute Care Health System

**DOI:** 10.1017/ash.2024.329

**Published:** 2024-09-16

**Authors:** Joan Ivaska, Milena Walker, Claire Jai, Marjorie Bessel

**Affiliations:** Banner Health; Banner Estrella Medical Center; Banner University Medical Center Phoenix

## Abstract

**Background:** Patients infected or colonized with Candida auris can serve as a transmission source for other patients. Screening patients for Candida auris colonization allows facilities to implement infection prevention and control measures and minimize the risk of transmission. The Centers for Disease Control and Prevention (CDC) recommends healthcare facilities perform three types of screening; admission screening in patients with specific risks, close contact screening of patients who overlap a confirmed positive case for 3 or more days or point prevalence surveillance if there is evidence of ongoing transmission within the facility. The CDC further recommends that patients being screened for Candida auris be maintained in transmission-based precautions while awaiting **Results:** In 2022-2023 there was ongoing transmission of Candida auris occurring in a community served by a large multi-state healthcare system. Close contact and point prevalence surveillance screening for both acute and non-acute healthcare facilities were implemented by the local health jurisdiction. **Methods:** A composite swab of the bilateral axilla and groin was used to screen close contacts of patients confirmed to be infected or colonized with Candida auris. Close contact was defined as having been on the same unit as the positive patient for 3 or more days while the patient was not in transmission-based precautions. Point prevalence surveillance was performed on all patients currently housed on units where close contact screen-positive patients resided. Potentially exposed patients who had been discharged were not screened. Patients were placed in contact transmission-based precautions until results were received. In 1657 patients in six acute care facilities were identified for Candida auris screening. 161 patients refused or were unable to be screened. Of the 1496 patients screened, 40 screened positive, demonstrating a 2.67% secondary attack rate. Of the 40 screen-positive patients, 5 were identified through point prevalence and 35 through close contact screening. **Conclusion:** Performing point prevalence surveillance in acute care facilities is operationally challenging and costly with little benefit in the prevention of Candida auris transmission. More robust collection and reporting of screening data is needed to inform surveillance protocols and prevention strategies specific to different healthcare settings. Limitations of this study include the lack of screening completion in discharged patients identified as close contact or point prevalence surveillance eligible. Additionally, some patients had a history of contact with healthcare facilities outside of this healthcare system, with unknown exposure risks or prevention strategies.

